# Natural extracts from fresh and oven‐dried winemaking by‐products as valuable source of antioxidant compounds

**DOI:** 10.1002/fsn3.697

**Published:** 2018-07-14

**Authors:** Lourdes Marchante, Sergio Gómez Alonso, María Elena Alañón, María Soledad Pérez‐Coello, María Consuelo Díaz‐Maroto

**Affiliations:** ^1^ Area of Food Technology Faculty of Chemical Sciences and Technologies University of Castilla‐La Mancha Ciudad Real Spain; ^2^ Area of Food Technology Regional Institute for Applied Scientific Research (IRICA) University of Castilla‐La Mancha Ciudad Real Spain

**Keywords:** antioxidant capacity, oven‐drying, phenolic profile, red winemaking by‐products

## Abstract

Winemaking by‐products are a natural source of antioxidant components; however, due to their highly perishable and seasonal nature, they may require a prior conservation step before being processed. Natural extracts from fresh and oven‐dried red grape agro‐industrial by‐products were obtained by ultrasound assisted extraction (UAE), using a hydroalcoholic solution as extracting solvent. Extracts were analyzed by HPLC‐DAD‐ESI‐MS, to know the feasibility of winemaking by‐products as natural sources of phenolic compounds, as well as the effect of the oven‐drying treatment on the phenolic composition. Oven‐drying at 45°C caused a significant decrease on the total phenolic content, which implied a reduction of the antioxidant capacity of the extracts. Also, it produced a decrease in total and individual flavan‐3‐ols, stilbenes, and flavonols, being greater in those extracts from stems. Respect to anthocyanins, which were only identified in grape pomace extracts, oven‐drying caused an important decrease, being the peonidin‐3‐O‐glucoside the more thermosensitive compound. Natural extracts from fresh or oven‐dried winemaking by‐products could be used in other food industries as a valuable source of phenolic compounds with antioxidant properties. However, further studies on other drying methods are required for addressing the preservation of phenolic compounds from winery by‐products successfully.

## 1 INTRODUCTION

Spain is the third world producer of wine, with 39.3 mhl (millions of hectoliters) in 2016 (OIV, Organisation Internationale de la Vigne et du Vin, 2016). It is well‐known that the vinification process generates high volumes of residues such as grape pomace, seeds, and stems, which should be adequately treated to avoid important environmental problems.

Nowadays, the use/revalorization of winemaking by‐products to obtain antioxidant and antimicrobial rich extracts, as well as functional compounds is the main challenge of several studies. Among winemaking residues, grape pomace presents a high content of phenolic compounds which are regarded as bioactive compounds due to their multiple functions (González‐Paramás, Esteban‐Ruano, Santos‐Buelga, De Pascual‐Teresa, & Rivas‐Gonzalo, 2004; Ribeiro, Ribani, Francisco, Soares, & Pontarolo, 2015). Generally, grape pomace represents 20–30% of the initial weight of grape (Rondeau, Gambier, Jolibert, & Brosse, 2013) and is characterized by a high residual total polyphenol content, depending on the grape variety or winemaking technique (Makris, Boskou, Andrikopoulos, & Kefalas, 2008).

Red grape pomace is mainly composed of seeds and skins, which are rich in flavonoids, although their profiles are different. Flavan‐3‐ols, monomeric and oligomeric forms, are found in seeds; while anthocyanins are exclusively present in red grape skins, which also have flavan‐3‐ols and flavonols (Gómez‐Alonso, García‐Romero, & Hermosín‐ Gutiérrez, 2007; Rodríguez Montealegre, Romero Peces, Chacón Vozmediano, Martínez Gascueña, & García‐Romero, 2006).

Other important winery by‐products are the grape stems, which are removed in the early stages of the winemaking process, and are not subjected to maceration phenomena. For this reason, the stems can retain their phenolic composition almost intact, being an important natural source of stilbenes, which are compounds with significant bioactive properties (Barros et al., 2014; Makris, Boskou, & Andrikopoulos, 2007; Ruiz‐Moreno et al., 2015; Spatafora, Barbagallo, Amico, & Tringali, 2013).

To obtain phenolic‐rich extracts, conventional extraction methods with organic solvents are commonly used. These extraction techniques are highly polluting, labor‐intensive, and time‐consuming, so the development of green alternative methods is required. The ultrasound assisted extraction (UAE) could be one of the most suitable techniques, as it is very simple to use, requires relatively inexpensive apparatus, reduces the volume of solvent, and allows to use green extraction solvents with a high efficiency due to the mechanical effects that it produces in the cellular structure of the matrix (Barba, Zhu, Koubaa, Sant′Ana, & Orlien, 2016).

The UAE has been used for the recovery of different bioactive components from fresh winemaking by‐products (Drosou, Kyriakopoulou, Bimpilas, Tsimogiannis, & Krokida, 2015; González‐ Centeno, Comas‐Serra, Femenia, Rosselló, & Simal, 2015; Piñeiro, Guerrero, Fernández‐Marin, Cantos‐Villar, & Palma, 2013), although in most cases a comprehensive study of the remaining polyphenolic composition of extracts has not been carried out. On the other hand, it is important to highlight the highly perishable and seasonal nature of winemaking by‐products. The drying treatment could be an effective method to increase their shelf life by slowing the development of microorganisms and preventing biochemical reactions that may modify the phenolic composition of those by‐products. Therefore, the objectives of this study were (1) the assessment of the feasibility of red winemaking by‐products (grape pomace, seed, and stems) from *Vitis vinifera* L. Cv. Tempranillo, as valuable and natural sources of polyphenols and (2) study the effect of the oven‐drying on the phenolic composition of the extracts. For these purposes, a green technology extraction such as ultrasonic‐assisted extraction with hydroalcoholic mixtures as extracting solvent was used. Phenolic‐rich extracts were characterized by HPLC‐DAD‐ESI‐MS, and chemical data were co‐related with the antioxidant capacity of the extracts.

## 2 MATERIALS AND METHODS

### 2.1 Fresh and oven‐dried samples

Among the raw materials studied were included grape pomace, seeds, and stems from *Vitis vinifera* L. Cv. Tempranillo. These winery by‐products were obtained from the Institute of Vine and Wine of Castilla‐La Mancha (IVICAM, Tomelloso, Ciudad Real, Spain). Samples were ground with addition of dry ice and cooling jacket at 0 ° C, using a crusher Stephan UMC5 (Stephan Food Service Equipment GMBH). This step is essential to get homogeneous samples with the same particle size (diameter less than 2 mm). Then, they were divided into two batches. One of them was conserved in a freezer at −20°C until their processing in fresh, and the remaining batch was immediately oven‐dried at 45°C during 17 hr for grape pomace, and during 19 hr for seeds and stems. The drying conditions were selected after trials had been conducted to achieve a percentage moisture content of <10% using the lowest temperature and shortest possible time. Oven‐dried samples were stored at room temperature until further processing and analysis.

The initial moisture content of the fresh samples was 64.3%, 53.6%, and 66.6% for grape pomace, seeds, and stems, respectively. While the moisture content of the dried samples was 6.0%, 5.0%, and 9.7%. The moisture content was determined (in duplicate) using a laboratory oven at 105°C.

### 2.2 Ultrasound assisted extraction

Ultrasound assisted extraction (UAE) was carried out in a QSONICA sonicator Q500 (53 CHURCG HILL RD. Newtown, CT, USA). To this end, 5 g of crushed sample was mixed with 20 ml of an hydroalcoholic solvent (44% of ethanol) in a beaker, operating at 20 KHz frequency, and 81% of output amplitude, with a duty cycle of 15‐s turn on and 5‐s off for an extraction period of 3 min. The beaker was introduced into a cooling bath at 4°C, and the extraction temperature was maintained at 20°C. Two extraction cycles were performed, and the mixtures were centrifuged at 7000 rpm for 5 min after extraction. The supernatants were collected and filtered under vacuum. These extracts were adjusted to the same volume (50 ml) and stored at −20°C in the dark until their analysis. Extractions from fresh and oven‐dried by‐products were performed in duplicate. UAE conditions were previously optimized by Response Surface Methodology (RSM). The optimization was realized with crushed oven‐dried grape pomace *Vitis vinifera* L. Cv. Tempranillo.

### 2.3 RSM experimental design

RSM was used to optimize the extraction conditions of total phenolic compounds (TPC) from oven‐dried grape pomace. A Central Composite Design was used to study the effect of two independent variables, ethanol content in the hydroalcoholic solvent (25–50%, v / v), and output amplitude (60–90%) on the response variable selected (TPC). The design consisted of 11 points, four (2^2^) factorial points, four axial points (2 × 2), and three repetitions at the central point for experimental error. The range and central point values of the two independent variables used are summarized in Table 1. To minimize the effects of unexpected variability in the responses, the experiments were run at random.

**Table 1:** RSM experimental (2^2^ design) and results obtained for response variable: TPC. Experimental and predicted values by the mathematical model are shown

Run	Ethanol content (%)	Output amplitude (%)	TPC (mg GAE/g)		Confidence interval, 95%
			Experimental value	Predicted value	
1	45	90	31.3	28.9	19.4–38.5
2	21	75	12.4	13.8	4.3–23.4
3	35	96	19.4	24.3	14.8–33.9
4	25	60	16.8	19.4	9.8–28.9
5	35	54	29.9	24.8	15.3–34.4
6	49	75	29.4	27.8	18.3–37.4
7	25	90	20.6	16.1	6.5–25.7
8	45	60	21.7	26.4	16.8–35.9
9	35	75	27.1	27.2	20.3–34.2
10	35	75	26.1	27.2	20.3–34.2
11	35	75	28.5	27.2	20.3–34.2

*Note*

TPC, total phenolic compounds.

Optimal extraction conditions were estimated by multiple linear regression (MLR) using Statgraphics Centurion XVI Version 16.1.17 (Statpoint Technologies, Warrenton, VA, USA). The residual standard deviation (RSD) and the coefficient of determination (*R* ^2^) were used to estimate the accuracy of the model.

### 2.4 Total phenolic content

To determinate the TPC of the extracts, the Folin–Ciocalteu method was followed (Singleton & Rossi, 1965). One hundred microliters (100 μl) of extract was mixed with 7.9 ml of Milli‐Q water and 500 μl of Folin‐Ciocalteu reagent. Then, 1.5 ml of sodium carbonate solution (5 g/L) in Milli‐Q water was added to the tube and the solution was shaken. The tubes were kept at room temperature, during 2 hr, and the absorbance was measured at 765 nm in a spectrophotometer (Helios, Thermo Spectronic, Cambridge, UK), versus a blank prepared without extract. All of the analyses were carried out in duplicate. The results were expressed as milligram of gallic acid equivalents per gram of dry weight sample (mg GAE/g DW), using gallic acid as standard.

### 2.5 Antioxidant activity determination

#### 2.5.1 DPPH assay

The DPPH assay was carried out using 1,1‐diphenyl‐2‐picrylhydrazyl as a stable radical (Brand‐Williams, Cuvelier, & Berset, 1995). One hundred microliters of different dilutions of extracts was mixed with 2.9 ml of a methanol DPPH radical solution (6.10^−5^ mM). Then, the decrease in absorbance was measured every minute for 25 min, at 515 nm, in a spectrophotometer (Helios, Thermo Spectronic). The zero was adjusted with methanol. A calibration curve (0.1–0.8 mM Trolox) was used to calculate the antioxidant activity of the extracts, and the results were expressed in mM Trolox equivalents per gram of dry weight sample (mM/mg DW).

#### 2.5.2 ABTS assay

The ABTS assay was carried out according to the method of Re et al. (1999). First, a 7 mM ABTS solution was mixed with a 2.45 mM potassium persulfate solution, to prepare the ABTS radical cation (ABTS˙^+^). The mixture was kept in the dark for 12–16 hr before use, and then, it was diluted with ethanol (1:90 v/v) to achieve an absorbance value of 0.7 (±0.02). Once ABTS˙^+^ working solution was obtained, 2 ml was mixed with 20 μl of diluted extracts. In the case of the blank, 20 μl of ethanol was used. Absorbance was measured in a spectrophotometer (Helios, Thermo Spectronic) at 734 nm, at 0 and 5 min of reaction. Readings at both times of reaction were used to calculate the percentage inhibition value for each extract. A calibration curve (0.1–0.8 mM Trolox) was used to calculate the antioxidant activity of the extracts, and the results were expressed in mM Trolox equivalents per gram of dry weight sample (mM/mg DW).

### 2.6 HPLC‐DAD–ESI–MSn analysis

#### 2.6.1 Flavan‐3‐ols, mean degree of polymerization (mDP), % galloylation, % prodelphinidines, and stilbenes

The first step on the analysis of flavan‐3‐ols (monomers, dimers, and polymeric proanthocyanidins) and stilbenes was a purification of the extracts using SPE on C18 cartridges (Sep‐pak Plus C18, Waters Corp., Milford, MA, USA; cartridges filled with 820 mg of adsorbent). After conditioning the SPE cartridge with 5 ml methanol and 5 ml water, 2 ml of each extract diluted with 12 ml of water was passed through them. Then, the cartridge was dried under reduced pressure and 15 ml methanol and 5 ml ethyl acetate were added sequentially to elute the flavan‐3‐ols. After the solvent was evaporated in a rotary evaporator (35°C), the residue was dissolved in 2‐ml methanol and stored at −18°C until its analysis.

Analysis of flavan‐3‐ol monomers, dimers, proanthocyanidins oligomers (condensed tannins), and stilbenes was carried out according to the previously described methodology (Lago‐Vanzela, Da‐Silva, Gomes, García‐Romero, & Hermosín‐Gutiérrez, 2011; Rebello et al., 2013). The analyses were performed in an Agilent 1200 series system equipped with a diode array detector (DAD; Agilent, Germany) and coupled to a mass spectrometry system AB Sciex 3200 Q TRAP (Applied Biosystems) operating in Multiple Reaction Monitoring (MRM) mode. Data processing was carried out with Analyst MSD software (version 1.5). Chromatographic separation was achieved on an Ascentis‐C18 column (4.6 × 150 mm; 2.7 μm particle; Supelco, Germany), thermostated at 16°C and with a flow rate of 0.3 ml/min.

Flavan‐3‐ol monomers, dimer procyanidins B1, B2, and B4, and stilbenes were quantified using the calibration curve of each standard. (+)‐Catechin, (−)‐epicatechin, (+)‐catechin gallate, (−)‐epicatechin gallate, (−)‐epigallocatechin gallate, and trans‐resveratrol‐glucoside were purchased from Sigma (Tres Cantos, Madrid, Spain). (+)‐Gallocatechin and (−)‐epigallocatechin from Phytolab (Vestenbergsgreuth, Germany). And procyanidins B1, B2, and B4 from Extrasynthese (Genay, France).The *trans* resveratrol‐3‐glucoside was transformed into its respective *cis* isomer using UV irradiation (366 nm during 5 min in quartz vials) with 25% Me OH solution of the *trans* isomer.

The mean degree of polymerization (mDP), % galloylation, and % prodelphinidines was calculated according to the procedures previously described by Kennedy and Jones (2001).

#### 2.6.2 Flavonols and hydroxycinnamic acids

To avoid the interferences that anthocyanins may cause in the chromatographic separation and identification of other phenolic compounds, those were separated from the extracts. Polymeric cation exchange SPE cartridges (PCX 500 mg, 6 ml; Bond Elut Plexa, Agilent) were used according to the method previously described by Castillo‐Muñoz, Gómez‐Alonso, García‐Romero, and Hermosín‐Gutiérrez (2007).

Extracts from stems and seeds have not anthocyanins, and thus, the separation of these compounds was not required. In this case, 2 ml of these extracts was taken to dryness in a miVac DUO concentrator operating at 35°C (Genevac Ltd., Ipswich, UK) and re‐solved in 1.5 ml of a methanol/water solution (20:80). Then, these were filtered (polyester membrane, 0.20 μm, Chromafil PET 20/25, Machery‐Nagel, Düren, Germany).

HPLC analysis of flavonols and hydroxycinnamic acid derivatives was performed on an Agilent 1100 Series system (Agilent, Germany). The system was equipped with a diode array detector (DAD) and a LC/MSD Trap VL electrospray ionization mass spectrometry (ESI‐MS/MS) detector connected in series. Extracts were injected into a reversed‐phase column Zorbax Eclipse XDB‐C18 (2.1 × 150 mm; 3.5 μm particle; Agilent), with a precolumn Zorbax Eclipse XDB‐C8 (2.1 × 12.50 mm; 5 μm particle; Agilent), themostatized at 40°C. Injection volume was 20 μl, flow rate was 0.16 ml/min, and the separation was achieved using a ternary mobile phase (Castillo‐Muñoz, Fernández‐González, Gómez‐Alonso, García‐Romero, & Hermosín‐Gutiérrez, 2009).

ESI‐MS/MS was used for identification, and DAD chromatograms at 360 nm and 320 nm were used for quantification of flavonols and hydroxycinnamic acids derivatives, respectively. Flavonol concentrations were expressed as equivalents of quercetin‐3‐glucoside (Extrasynthese, Genay, France) (μg/g of dry weight sample), while hydroxycinnamic acids were not found in the samples.

#### 2.6.3 Anthocyanins and derived compounds

HPLC analysis of anthocyanins was carried out on an Agilent 1100 Series system (Agilent, Germany), according to the methodology previously described by Rebello et al. (2013). The system was equipped with a diode array detector (DAD) and a LC/MSD Trap VL electrospray ionization mass spectrometry (ESI‐MS/MS) detector connected in series. The separation was realized in the same chromatographic column, themostatized at 40°C, used for flavonols. In this case, injection volume was 10 μl and the flow rate was 0.19 ml/min.

ESI‐MS/MS was used for identification. DAD chromatograms at 520 nm were used for quantification, and anthocyanin concentrations were expressed as equivalents of malvidin‐3‐glucoside (Phytolab) (μg/g of dry weight sample). Data processing was performed with Agilent ChemStation software (version B.01.03).

### 2.7 Statistical analysis

In order to identify statistically significant differences between TPC, antioxidant activity and phenolic compounds of different extracts, analysis of variance (ANOVA) and Student–Newman–Keuls test were applied to the analytical data. In the case of anthocyanins, an independent sample test was applied. Spearman's correlation coefficient was used to study the contribution of each phenolic compound in the antioxidant activity. The statistical package used was the IBM SPSS statistics v.22.0 for Windows.

## 3 RESULTS AND DISCUSSION

### 3.1 Optimization of the extraction conditions

UAE process is affected by numerous parameters such as solvent nature, output amplitude, extraction time, solid‐liquid ratio, etc. For this reason, different trials were performed according to the experience of the research group and to the UAE conditions of the bibliography (Carrera, Ruiz‐Rodriguez, Palma, & Barroso, 2012; Drosou et al., 2015; Paini et al., 2016). for optimize the extraction process. Trials were realized with oven‐dried grape pomace *Vitis vinifera* L. Cv. Tempranillo, and the TPC was used as a measure of the efficiency of the UAE. First, the amount of sample and the volume of extracting solvent were selected according to the probe tip diameter (12 mm) and to the operation manual of the QSONICA Q500. In order to be able to work with the smallest volume of extracting solvent (20 ml), a sample quantity of 5 g was selected. This sample quantity allowed to obtain a low mass/volume ratio and consequently an increase in the recovery of total polyphenols, without losing sensitivity in the detection of the individual extracted phenolic compounds.

Once the amount of sample was fixed, two extraction times (3 and 6 min) were tested at different output amplitudes and percentages of ethanol in the extracting solvent. Ethanol and water were selected as extracting solvents because they are the most suitable for further applications of the extracts in food industry. No differences were found between 3 and 6 min of time extraction; therefore, the extraction time selected was 3 min.

To optimize the percentage of ethanol in the extracting solvent and the output amplitude, an experimental design was performed. Percentage of alcohol and output amplitude were chosen as independent variables, and a Central Composite Design was used to carry out the optimization of both variables using the TPC as response variable. The levels of both variables were fixed between 25 and 50% for ethanol content in the extracting solvent and between 60 and 90% for output amplitude. Both intervals were selected according to the results obtained in a preliminary study in which an increase in the extraction of total polyphenols was observed as the output amplitude increased, mainly at low percentages of ethanol in the extracting solvent. However, when increasing the percentage of ethanol, no differences were obtained between 90 and 100% of output amplitude. In addition, an output amplitude of 100% involved excessive heating of the sample.

The experimental design conditions and the results obtained for TPC are shown in Table 1. It can be observed that experimental values were similar to those predicted. Also, TPC values obtained from the three central point of design pointed out low experimental errors. Table 2 shows the quadratic model obtained. The proposed model explained 70.45% of the variability in TPC (*R* ^2^), the RSD was 2.68, and the Durbin‐Watson statistic (DW) indicated that there was no significant correlation based on the order of conducting the experiments (*p *≥* *0.05). According to the statistical model obtained, the optimal conditions for maximum extraction of total polyphenols were 44% of ethanol in the hydroalcoholic solvent and an output amplitude of 81%. The experimental results for TPC were compared with that predicted by the statistical model with a 95% confidence interval. With the optimal conditions, the model predicted a value of 29.3 mg GAE/g for the response variable, similar to the experimental value reached under the same conditions (28.5 mg GAE/g). Under the optimized conditions, extractions from fresh and oven‐dried by‐products were performed by means of UAE in duplicate.

**Table 2:** Statistics and parameters of the mathematical model

Constant	27.23
Ethanol	9.91
Amplitude	−0.37
Ethanol x Ethanol	−6.40
Ethanol x Amplitude	2.91
Amplitude x Amplitude	−2.66
*R* ^2^	0.704
RSD	2.68
DW statistic	1.99 (*p *=* *0.43)

### 3.2 Antioxidant capacity and total polyphenol content of the extracts

Table 3 shows the mean values of antioxidant capacity (ABTS and DPPH) and total phenolic content of the extracts from the three red winemaking by‐products, together with the changes produced by the heat treatment (oven‐drying at 45°C). Fresh samples had similar contents of total polyphenols and did not present significant statistical differences. However, the values obtained for the antioxidant capacity were higher in those extracts obtained from fresh grape pomace and stems. These results seemed to suggest that despite the similar total phenol content, the phenolic profile of extracts from diverse winemaking by‐products should be different which could explain the different antioxidant capacity values observed.

**Table 3:** Mean values and standard deviations of antioxidant capacity and total phenol content of fresh and oven‐dried red winemaking by‐product extracts (*n *= 2)

	ABTS^a^	DPPH^a^	TPC^b^
Fresh grape pomace	0.156^c^ ± 0.031	0.273^c^ ± 0.044	39.5^b^ ± 3.1
Fresh stems	0.126^b,c^ ± 0.013	0.259^c^ ± 0.011	35.7^b^ ± 0.8
Fresh seeds	0.107^b^ ± 0.002	0.189^b^ ± 0.011	35.3^b^ ± 4.0
Oven‐dried grape pomace	0.051^a^ ± 0.009	0.136^a^ ± 0.007	28.5^a^ ± 0.6
Oven‐dried stems	0.051^a^ ± 0.001	0.089^a^ ± 0.001	17.2^a^ ± 0.9
Oven‐dried seeds	0.056^a^ ± 0.011	0.096^a^ ± 0.017	20.1^a^ ± 4.0

*Notes*

TPC, total phenolic compounds.

^a,b,c^Different letters in the same column indicate statistical significant differences between extracts (Student–Newman–Keuls test, α = 0.05).

a ABTS and DPPH: antioxidant capacity expressed as mmol of Trolox equivalents per gram of dry weight sample.

b TPC: total phenol content expressed as milligram of gallic acid equivalents per gram of dry weight sample.

On the other hand, it was evidenced that oven‐drying at 45°C caused a significant decrease on the total phenolic content which implied a reduction in the antioxidant capacity of the extracts. Similar results have been previously obtained during oven‐drying at higher temperatures (55–60°C) in red grape pomace and seeds (Del Pino‐García, González‐SanJosé, Rivero‐Pérez, García‐Lomillo, & Muñiz, 2017).

### 3.3 Phenolic profile of the extracts. Effect of the oven‐drying

Table 4 shows the individual and total concentrations (μg/g dry weight sample) of flavan‐3‐ols, mean degree of polymerization, % of galloylation, and % of prodelphinidines in the fresh and oven‐dried red winemaking by‐product extracts. The total amount of flavan‐3‐ols ranged from 696.9 to 3429.0 μg/g for extracts from oven‐dried stems and fresh seeds, respectively. Fresh seed extracts present the major quantities of total flavan‐3‐ols, following by fresh grape pomace and stem extracts. The total quantity of flavan‐3‐ols decreased considerably during oven‐drying. The highest losses of flavan‐3‐ols occurred in the stems, while seeds were the winemaking by‐products less affected by oven‐drying.

**Table 4:** Individual and total concentrations (μg/g dry weight sample) of flavan‐3‐ols, mean degree of polymerization, % of galloylation and % of prodelphinidines in natural extracts from fresh and oven‐dried red winemaking by‐products (*n *= 2)

Flavan‐3‐ols	Fresh stems	Oven‐dried stems	Fresh seeds	Oven‐dried seeds	Fresh grape pomace	Oven‐dried grape pomace
(+)‐Catechin	771.0^d^ ± 48.4	167.0^a^ ± 31.9	777.4^d^ ± 14.3	408.3^b^ ± 20.6	640.7^c^ ± 71.6	197.5^a^ ± 32.3
(−)‐Epicatechin	44.0^a^ ± 4.6	12.9^a^ ± 3.4	347.8^e^ ± 8.9	187.8^c^ ± 26.3	289.8^d^ ± 6.9	117.5^b^ ± 20.3
(+)‐Gallocatechin	1.4^b^ ± 0.1	0.3^a^ ± 0.1	1.4^a^ ^.b^ ± 0.1	0.5^a^ ^.b^ ± 0.1	1.1^.b^ ± 0.30	0.4^a^ ^.b^ ± 0.2
(−)‐Epigallocatechin	6.9^c^ ± 0.5	0.7^a^ ± 0.1	0.0^a^ ± 0.0	0.0^a^± 0.0	2.0^b^ ± 0.7	0.0^a^ ± 0.0
(+)‐Catechin gallate	40.7^d^ ± 1.8	9.3^a^ ± 2.3	22.7^b^ ± 0.4	14.4^a^ ± 2.8	31.2^c^ ± 2.7	13.2^b^ ± 0.22
(−)‐Epicatechin gallate	41.2^d^ ± 1.8	9.4^a^ ± 2.3	23.0^b^ ± 0.4	14.5^a^ ± 2.8	31.6^c^ ± 2.7	13.4^a^ ± 0.2
(−)‐Epigallocatechin gallate	9.1^b^ ± 2.1	0.1^a^ ± 0.0	0.2^a^ ± 0.3	0.1^a^ ± 0.1	0.0^a^ ± 0.0	0.0^a^ ± 0.0
Procyanidin B1	1225.2^d^ ± 27.4	321.5^b^ ± 51.7	439.0^c^ ± 16.7	223.9^a^ ± 27.9	414.8^c^ ± 33.0	154.0^a^ ± 17.9
Procyanidin B2	54.6^a^ ± 5.6	21.2^a^ ± 3.8	571.9^d^ ± 3.6	298.4^c^ ± 16.2	625.5^e^ ± 2.3	242.4^b^ ± 30.1
Procyanidin B4	0.0^a^ ± 0.0	0.0^a^ ± 0.0	252.1^e^ ± 5.8	119.7^c^ ± 12.3	188.5^d^ ± 7.5	61.7^b^ ± 5.1
Procyanidin (Unknown 1)	181.5^d^ ± 3.1	29.5^a^ ± 5.5	230.7^e^ ± 4.7	100.5^b^ ± 11.0	125.8^c^ ± 6.1	39.2^a^ ± 4.3
Procyanidin (Unknown 2)	113.1^c^ ± 5.7	30.6^a^ ± 5.1	56.4^b^ ± 1.2	27.9^a^ ± 1.9	51.1^b^ ± 3.1	21.0^a^ ± 2.5
Galloylated dimers	155.2^c^ ± 8.1	54.1^a^ ± 12.0	191.4^d^ ± 1.8	97.0^b^ ± 7.9	253.7^e^ ± 16.0	87.8^b^ ± 6.5
Monomer glycosides	124.8^b^ ± 8.0	40.6^a^ ± 5.8	515.2^e^ ± 7.5	283.1^d^ ± 10.5	185.9^c^ ± 29.4	70.1^a^ ± 7.9
Monomers^a^	1006.4^c.d^ ± 61.7	233.5^a^ ± 44.3	1670.5^e^ ± 30.2	898.1^c^ ± 60.8	1159.7^d^ ± 98.6	402.6^b^ ± 61.0
Dimers^a^	867.8^d^ ± 25.0	229.2^a^ ± 39.2	873.7^d^ ± 11.6	435.2^c^ ± 38.8	832.5^d^ ± 7.4	304.0^b^ ± 33.3
Total Oligomers^a^	12751.5^e^ ± 212.5	3254.1^a^ ± 229.3	9820.1^b^ ± 816.3	4324.8^a^ ± 487.3	9491.3^b^ ± 12.5	409.0^a^ ± 31.7
Total flavan‐3‐ols	2768.6^d^ ± 112.1	696.9^a^ ± 124.0	3429.0^e^ ± 52.9	1776.2^c^ ± 140.0	2841.6^d^ ± 85.7	1018.0^a^ ± 127.6
mDP	4.7^a^ ± 0.3	4.8^a^ ± 0.2	5.8^a^ ± 0.4	5.5^a^ ± 0.6	6.1^a^ ± 0.2	5.3^a^ ± 0.0
% Galloylation	3.7^a^ ± 0.0	3.4^a^ ± 0.2	6.8^b^ ± 0.6	7.6^b^ ± 0.5	7.7^b^ ± 0.3	8.5^b^ ± 0.9
% Prodelphinidines	45.7^a^ ^.b^ ± 1.4	42.9^a^ ± 1.3	50.2^b^ ± 1.0	49.4^b^ ± 2.9	48.8^b^ ± 0.4	49.5^b^ ± 1.9

*Notes*

^a,b,c,d,e^Different letters in the same row indicate statistical significant differences between extracts (Student–Newman–Keuls test, α = 0.05).

a Quantified as catechin equivalents.

In all samples, the main monomers were (+)‐catechin and its isomer (−)‐epicatechin. Extracts obtained from fresh samples present major concentration of (+)‐catechin, mainly those from seeds (777.4 μg/g) and stems (771.0 μg/g). While (−)‐epicatechin appeared in greater quantity in fresh seeds (347.8 μg/g) and grape pomace (289.8 μg/g) extracts. Both isomers decreased during oven‐drying. Other monomers identified were (+)‐gallocatechin, (−)‐epigallocatechin, (+)‐catechin gallate, (−)‐epicatechin gallate, and (−)‐epigallocatechin gallate, which are found in low concentrations in grapes (Gagné, Saucier, & Gény, 2006; Souquet, Cheynier, Sarni‐Manchado, & Moutounet, 1996).

Respect to oligomers, procyanidin B1 was the most abundant flavan‐3‐ol in the extracts of fresh stems (1222.5 μg/g), which was in good agreement with those observations previously realized by González‐Centeno et al. (2012). However, its content decreased considerably during oven‐drying. On the other hand, procyanindin B2 was the main dimer in seed and grape pomace extracts. This compound was described as the main procyanidin dimer in seeds and grape pomace from different grape varieties (González‐Paramás et al., 2004).

Although the skin flavan‐3‐ols have a higher degree of polymerization than that from seeds (Labarbe, Cheynier, Brossaud, Souquet, & Moutounet, 1999), the results obtained in mean degree polymerization (mDP) did not show significant differences between extracts. Taking into account that extracts were obtained from winery by‐products, their residual quantities in flavan‐3‐ols will depend on factors such as winemaking conditions (Makris et al., 2007; Monagas, Gómez‐Cordovés, Bartolomé, Laureano, & Ricardo da Silva, 2003).

Seed and grape pomace extracts were richer in galloylated flavanols and prodelphinidines than extracts of stems, although the quantity of flavanols of grape pomace is totally conditioned by its proportion in seeds. It has been reported in bibliography that galloylated flavanols have an antioxidant activity higher than their nongalloylated homologues (Plumb, de Pascual‐Teresa, Santos‐Buelga, Cheynier, & Williamson, 1998).

Another group of important phenolic compounds are stilbenes. Their presence in plants is related with resistance to certain fungi such as *Botrytis cinerea*, or to other causes of stress, such as ultraviolet irradiation (Langcake & Pryce, 1977). These compounds have also gained significant attention because of their high antioxidant activity (Aziz, Kumar, & Ahmad, 2003). Table 5 displays the individual and total concentrations (μg/g dry weight sample) of stilbenes in the fresh and oven‐dried red winemaking by‐product extracts.

**Table 5:** Individual and total concentrations (μg/g dry weight sample) of stilbenes in natural extracts from fresh and oven‐dried red winemaking by‐products (*n *= 2)

Stilbenes	Fresh stems	Oven‐dried stems	Fresh seeds	Oven‐dried seeds	Fresh grape pomace	Oven‐dried grape pomace
t‐Resveratrol‐glucoside	3.44^d^ ± 0.52	0.63^a,b^ ± 0.26^b^	1.63^c^ ± 0.05	1.18^b,c^ ± 0.17	0.65^a,b^ ± 0.12	0.25^a^ ± 0.05
c‐Resveratrol‐glucoside	0.84^d^ ± 0.03	0.15^a,b^ ± 0.09^b^	0.30^c^ ± 0.02	0.17^b^ ± 0.02	0.10^a,b^ ± 0.02	0.03^a^ ± 0.00
Total Stilbenes	4.28^c^ ± 0.49	0.79^a,b^ ± 0.35	1.92^b^ ± 0.03	1.35^a,b^ ± 0.19	0.75^a,b^ ± 0.14	0.28^a,b^ ± 0.06

*Note*

^a,b,c,d^Different letters in the same row indicate statistical significant differences between extracts (Student–Newman–Keuls test, α = 0.05).

In all extracts, only the 3‐glycosylated form of resveratrol was found, with the *trans* isomer as majority. Extracts from fresh stems had the greatest amount of total stilbenes, highlighting their high quantity in *trans*‐resveratrol‐glucoside. Previous studies had revealed the higher content of resveratrol in stems than in seeds or skins (Cho et al., 2003). Stilbenes store at high levels in grape leaves and stems, occurring at significantly lower quantities in grapes (Langcake, 1981; Langcake & Pryce, 1977). Oven‐drying treatment produced a decrease in stilbenes in all the samples, being more important in the stem extracts.

Flavonols, another important group of phenolic compounds, were only detected and quantified in extracts of fresh and oven‐dried grape pomace and stems. Individual and total concentrations (μg/g dry weight sample) of flavonols are shown in Table 6. Extracts obtained from fresh stems presented the highest levels of total flavonols (385.69 μg/g), although only three glycosylated derivatives of quercetin (quercetin‐3‐glucuronide, quercetin‐3‐glucoside, and quercetin‐3‐O‐rutinoside) were detected. Among them, glucuronide derivative was the most abundant. In extracts of grape pomace the quercetin‐3‐glucuronide was not found; however, another four flavonols were identified, such as laricitrin‐3‐glucoside, kaempferol‐3‐glucoside, isorhamnetin‐3‐glucoside, and syringetin‐3‐glucoside. The oven‐drying treatment produced a significant decrease of total flavonols in both samples, grape pomace and stems, with losses in the amounts of all identified flavonols. These losses were greater in the stem extracts, as it was in the case of stilbenes and flavan‐3‐ols, showing the strong influence of the intrinsic characteristics of the sample on the behavior of different chemical compounds during drying (De Torres, Schumacher, Alañón, Pérez‐Coello, & Díaz‐Maroto, 2015).

**Table 6:** Individual and total concentrations (μg/g dry weight sample) of flavonols in natural extracts from fresh and oven‐dried red winemaking by‐products (*n *= 2)

Flavonols	Fresh stems	Oven‐dried stems	Fresh grape pomace	Oven‐dried grape pomace
Quercetin‐3‐glucuronide	299.70^c^ ± 46.76	93.80^b^ ± 5.26	0.00^a^ ± 0.00	0.00^a^ ± 0.00
Quercetin‐3‐glucoside	65.91^c^ ± 8.39	19.72^a^ ± 1.41	37.82^b^ ± 0.04	20.81^a^ ± 0.68
Quercetin‐3‐O‐rutinoside	20.09^c^ ± 3.18	7.85^b^ ± 0.26	2.56^a^ ± 0.53	1.13^a^ ± 0.04
Laricitrin‐3‐glucoside	0.00^a^ ± 0.00	0.00^a^ ± 0.00	19.17^c^ ± 0.05	10.88^b^ ± 1.09
Kaempferol‐3‐glucoside	0.00^a^ ± 0.00	0.00^a^ ± 0.00	8.14^c^ ± 0.01	5.34^b^ ± 0.17
Isorhamnetin‐3‐glucoside	0.00^a^ ± 0.00	0.00^a^ ± 0.00	6.58^a^ ± 0.03	4.13^b^ ± 0.38
Syringetin‐3‐glucoside	0.00^a^ ± 0.00	0.00^a^ ± 0.00	17.76^c^ ± 0.17	12.17^b^ ± 0.29
Total flavonols	385.69^b^ ± 58.34	121.37^a^ ± 6.93	92.04^a^ ± 0.71	54.46^a^ ± 0.40

*Note*

^a,b,c^Different letters in the same row indicate statistical significant differences between extracts (Student–Newman–Keuls test, α = 0.05).

As expected, the anthocyanins were only identified in the extracts of fresh and oven‐dried grape pomace. Table 7 displays the individual and total concentrations (μg/g dry weight sample). The main anthocyanins were malvidin‐3‐*O*‐glucoside and its coumaroyl and caffeoyl derivatives, as grape pomace was obtained from *Vitis vinifera* L. Cv. Tempranillo. Oven‐drying caused an important decrease in the total anthocyanins, being the compounds more affected petunidin‐3‐*O*‐glucoside, malvidin‐3‐*O*‐glucoside, and peonidin‐3‐*O*‐glucoside. Indeed, the occurrence of this latter anthocyanin was not detected in oven‐dried grape pomace.

**Table 7:** Individual and total concentrations (μg/g dry weight sample) of anthocyanins in natural extracts from fresh and oven‐dried red grape pomace (*n *= 2)

Anthocyanin	Fresh grape pomace	Oven‐dried grape pomace
Petunidin‐3‐O‐glucoside	52.27^b^ ± 0.65	5.61^a^ ± 0.25
Peonidin‐3‐O‐glucoside	8.27 ± 0.10	nd
Malvidin‐3‐O‐glucoside	433.13^b^ ± 13.91	79.92^a^ ± 0.74
Delphinidin‐3‐O‐caffeoylglucoside	10.42^b^ ± 0.83	2.38^a^ ± 0.05
Petunidin‐3‐O‐acetylglucoside	8.71^b^ ± 0.34	1.62^a^ ± 0.09
Petunidin‐3‐O‐caffeoylglucoside	13.52^b^ ± 0.61	3.14^a^ ± 0.05
Delphinidin‐3‐O‐coumaroylglucoside	57.50^b^ ± 1.13	9.01^a^ ± 0.35
Malvidin‐3‐O‐acetylglucoside	45.84^b^ ± 1.16	9.42^a^ ± 0.00
Peonidin‐3‐O‐caffeoylglucoside	7.20^b^ ± 0.35	2.01^a^ ± 0.03
Cyanidin‐3‐O‐coumaroylglucoside	9.68^b^ ± 0.21	2.14^a^ ± 0.08
Malvidin‐3‐O‐caffeoylglucoside	124.57^b^ ± 7.25	40.10^a^ ± 0.35
Petunidin‐3‐O‐coumaroylglucoside	68.63^b^ ± 1.81	16.08^a^ ± 0.48
Malvidin‐3‐O‐coumaroylglucoside cis‐	8.39^b^ ± 0.27	1.86^a^ ± 0.02
Peonidin‐3‐O‐coumaroylglucoside	23.47^b^ ± 0.78	6.12^a^ ± 0.19
Malvidin‐3‐O‐coumaroylglucoside trans‐	363.06^b^ ± 10.19	113.36^a^ ± 2.90
Total anthocyanins	1234.68^b^ ± 39.61	292.77^a^ ± 5.07

*Notes*

Nd, no detected.

^a,b^Different letters in the same row indicate statistical significant differences between extracts (Student–Newman–Keuls test, α = 0.05).

### 3.4 Correlation between antioxidant capacity and phenolic compounds of the extracts

A correlation analysis, between antioxidant values and phenolic composition, was performed in order to determine the possible contribution of the individual phenolic compounds in the antioxidant capacity of the extracts. Table 8 shows those phenolic compounds with stronger correlations.

**Table 8:** Spearman′s correlation matrix between antioxidant capacity values and the concentration of individual and total phenolic compounds

	ABTS	DPPH
(+)‐Catechin	0.776**	0.776**
(+)‐Gallocatechin	0.783**	0.797**
(−)‐Epigallocatechin	0.631*	0.728**
(+)‐Catechin gallate	0.881**	0.860**
(−)‐Epicatechin gallate	0.881**	0.860**
Procyanidin B1	0.825**	0.860**
Procyanidin (Unknown 1)	0.720**	0.678*
Procyanidin (Unknown 2)	0.797**	0.881**
Galloylated dimers	0.846**	0.783**
Flavan‐3‐ol monomers^a^	0.734**	0.699*
Flavan‐3‐ol dimers^a^	0.769**	0.727**
Flavan‐3‐ol total oligomers^a^	0.825**	0.811**
Total Flavan‐3‐ols	0.727**	0.713**
*trans*‐Resveratrol‐glucoside	0.497*	0.594*

*Notes*

^a^Quantified as catechin equivalents.

Significant correlation at the **0.01 and *0.05 level.

Positive correlations were observed between antioxidant activity and some flavan‐3‐ols, as (+)‐catechin (C), (+)‐gallocatechin (GC), (−)‐epigallocatechin (EGC), (+)‐catechin gallate (CG), (−)‐epicatechin gallate (ECg), procyanidin B1, two unknown procyanidins, and galloylated dimers. These compounds seemed to be the main contributors to the overall antioxidant capacity of extracts from winemaking by‐products. Consequently with our results, previous studies also indicated the strong antioxidant capacity of the total flavan‐3‐ols (Del Pino‐García et al., 2017; Villaño, Fernández‐Pachón, Moyá, Troncoso, & García‐Parrilla, 2007). On the other hand, the remarkable presence of (+)‐catechin and (−)‐epicatechin in grape pomace and stems have been previously correlated with higher antioxidant capacities of those samples (Alonso, Domínguez, Guillén, & Barroso, 2002).

In the extracts tested, the correlation of monomers with the antioxidant activity was as follows: CG > ECg > GC > C > EGC This correlation order was in good agreement with the evidences previously reported by Guo et al. (1999), and Nanjo et al. (1996). These authors pointed out that the presence of a gallate group in position 3′ and the occurrence of hydroxyl groupin position 5′of ring B play an important role to quench free radicals.

Dimers seemed to also play an important role in the antioxidant activity of extracts. The correlation between the procyanidin B1 content and antioxidant capacity had already been highlighted in grape seed extracts (Guendez, Kallithraka, Makris, & Kefalasa, 2005). The high values of antioxidant activity of procyanidin B1 have been attributed to the number of available hydroxyl groups (Alonso et al., 2002).

Another compound correlated to the antioxidant capacity of the extracts was *trans*‐resveratrol‐glucoside, although this compound was found at lower concentrations than flavan‐3‐ols. This compound has demonstrated DPPH scavenging capacity in other studies (Wei, Zhao, Li, & Xue, 2016), while its isomer, *cis*‐resveratrol‐glucoside showed less efficiency in the mechanism of transfer of an atom of hydrogen to the free radicals (Mikulski, Górniak, & Molski, 2010).

## 4 CONCLUSIONS

Ultrasound assisted extraction allows to obtain natural phenolic‐rich extracts from winemaking by‐products which could be used in other food industries, as a valuable source of antioxidant components. The optimal conditions for maximum extraction of total polyphenols were 44% of ethanol in the hydroalcoholic solvent and an output amplitude of 81%.

However, due to their highly perishable and seasonal nature, winemaking by‐products require a prior conservation step before being processed. In this regard, oven‐drying at 45°C caused a significant decrease on the total and individual phenolic components, which implied a reduction in the antioxidant capacity of the extracts. The changes observed during the heat treatment depended on the intrinsic characteristics of the raw materials and their phenolic composition. Further studies on other alternative drying methods would be of great value for the recovery of polyphenols from the winemaking by‐products.

## CONFLICT OF INTEREST

The authors declare that they do not have any conflict of interest.

## ETHICAL STATEMENTS

This study does not involve any human or animal testing, and an informed consent was obtained from all study contributors.
